# Neutrophils in the tumor microenvironment – when a company becomes a crowd

**DOI:** 10.1038/s41423-024-01147-9

**Published:** 2024-03-08

**Authors:** Zvi G. Fridlender, Zvi Granot

**Affiliations:** 1 Faculty of Medicine, Hebrew University of Jerusalem, Jerusalem, Israel; 2 Institute of Pulmonary Medicine, Hadassah-Hebrew University Medical Center, 91120 Jerusalem, Israel; 3 Department of Developmental Biology and Cancer Research, Institute for Medical Research Israel Canada, Faculty of Medicine, Hebrew University, 91120 Jerusalem, Israel

**Keywords:** Tumour immunology, Neutrophils

Traditionally, neutrophils are perceived as terminally differentiated cells with defined functions and limited plasticity. It is commonly believed that circulating, marginating and tissue-infiltrating neutrophils belong to the same homogeneous population and are capable of switching between these locations. Neutrophil research has stagnated since the early 1980s, maintaining a narrow and simplistic view of their capabilities. Over the past two decades, substantial advances in our understanding of neutrophils have revealed them to be a plastic and diverse population of cells [1, 2]. Several seminal studies have challenged the conventional perception of neutrophils, particularly in the context of cancer, where their role has been hotly debated. These studies demonstrated that neutrophils can exert either pro- or antitumor effects [3]. This controversy was ultimately resolved with the recognition that neutrophils constitute a heterogeneous population, exemplified by the discovery of two distinct tumor-associated neutrophil subsets, N1 and N2 with antitumor and protumor properties, respectively [4]. Moreover, the phenotype of neutrophils is influenced by molecular cues within the tumor microenvironment, contributing to a broader “immunosuppressive switch” that characterizes tumor progression [5]. In addition to these findings, a unique subset of neutrophils with lower density, termed low-density neutrophils (LDNs), was identified in cancer. LDNs differ from normal-density neutrophils (NDNs) and encompass at least two subpopulations: mature and immature LDNs. Furthermore, studies have shown that neutrophils can transition between the NDN and LDN states, underscoring their remarkable plasticity [2]. Importantly, while the plasticity of neutrophils initially gained recognition in the context of cancer, its importance extends to various clinical scenarios including chronic inflammation and infectious diseases [1]. Here we highlight the broad relevance and importance of understanding the diverse nature of neutrophils beyond their traditional roles.

**Fig. 1:**
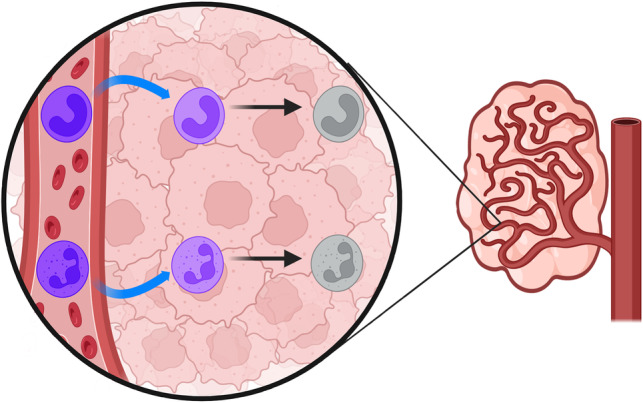
Source of tumor-associated neutrophils. Circulating mature and immature neutrophils are recruited to tumors, where they differentiate into T1 and T2 TANs. T1 and T2 further differentiate to form the T3 subset

Although substantial advances have been made in understanding neutrophils in recent decades, numerous important questions remain regarding the cellular origins and diversity of tumor-associated neutrophils (TANs). Specifically, the source of mature and immature LDNs and their infiltration into tumors, as well as the phenotypic changes they undergo within the tumor microenvironment, including the origin of N2 neutrophils, remain unresolved. A recent study by Ng et al. [6] elegantly addressed some of these questions, considerably advancing our comprehension of neutrophil biology in cancer. By employing state-of-the-art methodologies, the authors investigated the developmental trajectory of heterogeneous neutrophils both in the circulation and within tumors. Additionally, they explored how the tumor microenvironment shapes the phenotype of neutrophils and modulates their lifespan, ultimately leading to the adoption of a protumoral phenotype in which neutrophils actively contribute to the establishment of an immunosuppressive tumor microenvironment. In their study, Ng and colleagues demonstrated that both mature and immature neutrophils originating from the bone marrow infiltrate tumors, providing crucial insights into the cellular origins of TAN. Upon entring the tumor, these subsets adopt distinct epigenetic and transcriptional programs, designated T1 and T2 (Fig. 1). Moreover, they observed further reprogramming within the tumor, leading to the convergence of both T1 and T2 into a novel, terminally differentiated population of TANs designated T3 (Fig. 1). The T3 subpopulation is characterized in mice by dcTRAIL-R1 expression, a significantly prolonged lifespan (more than 5 days) and proangiogenic and protumoral functions. Notably, several features of the T3 population identified in this study, such as extended survival and increased VEGF expression, align with characteristics previously attributed to N2 neutrophils.

While these discoveries were made within the context of cancer research, they are potentially applicable to a broader spectrum of chronic inflammatory diseases and conditions, establishing several fundamental principles in neutrophil biology:

First, they unequivocally highlight the heterogeneity of circulating neutrophils originating from the bone marrow. Contrary to previous assumptions, these findings underscore the notion that circulating neutrophils are not uniform, terminally differentiated cells with a single fate. Instead, upon migrating into tissues, they respond to specific microenvironmental cues and undergo dynamic changes at the epigenetic and transcriptional levels, which instruct new and diverse functions. In essence, circulating neutrophils, akin to monocytes, can be regarded as cells in a state of ongoing differentiation within their destined tissues.

Second, this research suggests that neutrophils from various sources may converge into a functionally homogenous type of tumor-associated neutrophils (TANs). This phenomenon implies a degree of plasticity and adaptability in neutrophil function, influenced by the local tissue microenvironment.

Finally, the debate surrounding neutrophil lifespan is addressed in this study. Contrary to the prevailing belief in their short-lived nature, the authors demonstrated that tissue-associated neutrophils, particularly those within the tumor microenvironment, can survive for extended periods, surpassing 5 days, as previously suggested [7]. This finding challenges traditional notions regarding neutrophil longevity and underscores the importance of considering context-specific factors in understanding neutrophil behavior.

**Fig. 2:**
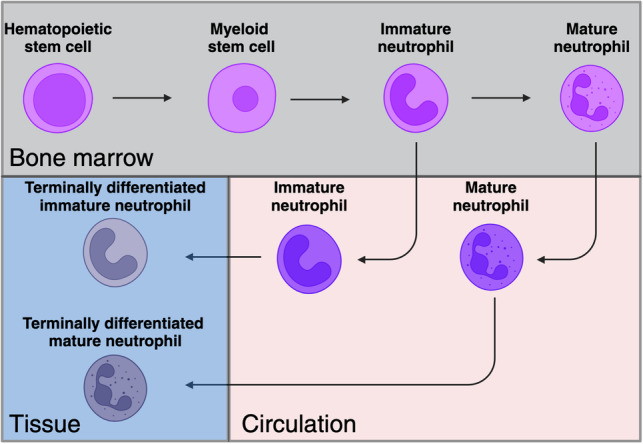
Suggested model for neutrophil subset maturation and differentiation. In health, the vast majority of neutrophils complete their maturation in the bone marrow before mobilization, whereas in pathology, neutrophils may leave the bone marrow prematurely. Ng et al. suggested that mature and immature neutrophils retain a degree of plasticity and can terminally differentiate when entering tissues

One of the most striking findings reported by Ng and colleagues is that the removal of factors driving the differentiation of T1/T2 to T3 does not result in a reversal of the phenotype [6]. On the one hand, this finding implies that neutrophils achieve terminal differentiation only upon further reprogramming within the tumor microenvironment (Fig. 2). Conversely, once this differentiation occurs, these neutrophils become impervious to alterations in the tumor milieu. This challenges the conventional belief that manipulating the tumor microenvironment can shift neutrophils from a pro- to an antitumor phenotype. Instead, it is suggested that modifying the tumor microenvironment, such as by inhibiting TGFβ signaling, would primarily impact newly arriving neutrophils rather than those that have already undergone intratumoral differentiation. This intriguing hypothesis has important clinical and therapeutic implications and warrants further investigation. Another crucial area for future research pertains to elucidating the specific functions of T1 and T2 neutrophils. While they may represent transitional phenotypes along the trajectory of deterministic reprogramming, it is conceivable that they exert distinct tumor-related functions, either pro- or antitumor, while residing in the tumor. These functions could significantly influence the overall contribution of neutrophils to tumor growth and progression, as the collective impact is determined by the cumulative functions of all neutrophil subsets at a given time point. Further exploration of these aspects is essential for a comprehensive understanding of neutrophil dynamics in the context of tumorigenesis. Another important research avenue arising from this study is the implementation of the concepts described here to human cancer patients. Finding the human parallels for T1-T3 and of the T3 marker suggested, dcTrail-R1, is imperative to further advance this line of research presented by Ng and colleagues.

In summary, the pivotal and intricate study conducted by Ng et al. unequivocally illustrates the heterogeneity and plasticity of neutrophils, shedding light on their tissue differentiation and challenging the notion of circulating mature neutrophils as terminally differentiated cells. This research marks an important milestone in our understanding of neutrophil biology within the framework of cancer. Moreover, it introduces novel concepts and ideas that may have broader implications for understanding neutrophil biology across various contexts.
